# The Analysis of Integration of Ideological Political Education With Innovation Entrepreneurship Education for College Students

**DOI:** 10.3389/fpsyg.2021.610409

**Published:** 2021-05-05

**Authors:** Xinyuan Zhao, Jinle Zhang

**Affiliations:** School of Marxism, Northwestern Polytechnical University, Xi’an, China

**Keywords:** positive entrepreneurship psychological quality, ideological and political education in colleges, innovation and entrepreneurship education, entrepreneurial self-efficacy, hierarchical regression

## Abstract

This study aims to analyze the integrated construction and application of ideological and political education (IPE) and innovation and entrepreneurship education (IEE) in colleges based on the positive psychological quality of entrepreneurship. 549 college students are selected for a questionnaire survey. The correlations between entrepreneurial psychological quality of college students, IEE in colleges, IPE, and entrepreneurial self-efficacy are analyzed with the Spearman correlation and linear regression. The hierarchical regression analysis is used to analyze the intermediary role of entrepreneurial self-efficacy in IEE, IPE in colleges, and entrepreneurial psychological quality of college students. The results show that the entrepreneurial psychological quality of college students is significantly different in gender, family location, and grade level (*p* < 0.05); the main channels of IPE and daily IPE have significant positive effects on the positive entrepreneurship psychological quality (*p* < 0.05), and have extremely significant positive effects on the entrepreneurial self-efficacy (*p* < 0.001); innovation and entrepreneurship course, innovation and entrepreneurship practices, innovation and entrepreneurship environment, and total score of IEE have significant positive correlations with positive entrepreneurship psychological quality and entrepreneurial self-efficacy of college students (*p* < 0.05); and the intermediary effect of entrepreneurial self-efficacy accounts for 33.49% on the IPE and entrepreneurial psychological quality, and 41.85% on IEE and entrepreneurial psychological quality. In short, IPE and IEE can effectively improve the positive psychological quality and self-efficacy of college students, and the joint construction of the two can have a more significant effect.

## Introduction

Innovation and entrepreneurship education (IEE), aiming at cultivating talents with basic entrepreneurial qualities and pioneering personalities, is to cultivate the entrepreneurial awareness, entrepreneurial spirit, and innovative entrepreneurial ability of students, and the high-quality innovative talents with innovative thinking and entrepreneurial ability by training the basic entrepreneurial skills (Hmieleski and Lerner, 2016; Sun, 2020; Yang, 2020). IEE in colleges has always been a matter of great concern to the Chinese government. The Ministry of Education stated in the *“Opinions on Promoting IEE in Colleges and Universities and Entrepreneurship Work for College Students”* that developing IEE in colleges and universities and actively encouraging college student to start up business is a major strategic measure for the education system to take deep learning and practice of the scientific development concept and serving the construction of an innovative country (Tritt et al., 2016). With the development of the diversified situation of social transformation in China, the pattern of Marxism occupying the main belief position is gradually broken, which makes entrepreneurship college students inevitably fall into the dilemma of lack of political beliefs and confused value orientations (Wang et al., 2019). Ideological and political theory course is the main way for IPE in colleges and universities in China, so it should keep the pace with times to ensure the political direction of strengthening moral education and cultivating people of the IEE, and lead the value orientation of the IEE (Wnuk et al., 2020). It is a crucial part of the new era to integrate the IPE with IEE for college students to train socialist builders and successors for the comprehensive development of moral, intellectual, physical, aesthetics, and labor skills.

Positive psychology is a kind of psychology that care about the excellent quality of people risen in the western psychology in the late 1990s, which was a symbolic movement that transforms psychological problems into positive powers of human body (Hoeppner et al., 2017). Based on the positive psychology, it believes that psychology should not only study the damages, defects, and injuries but also research the powers and excellent qualities; not only repair and compensate for damages and defects but also explore the potential and power of human beings; not only be the science about disease or health but also be the science about work, education, love, growth, and entertainment (Li et al., 2020). For college students in entrepreneurship, it is not enough to learn rich theoretical knowledge of entrepreneurship, and they have to pay more attention to their own mental health, obtain positive entrepreneurial intentions and tenacious entrepreneurial will, so that the entrepreneurial ideas can be invested in the entrepreneurship practice (Wagemans et al., 2016). Therefore, the positive psychological quality advocated by the positive psychology can better help college students face some difficulties in entrepreneurship, achieve successful entrepreneurship and life value, and acquire the subjective well-being (Fodor and Pintea, 2017). Therefore, it intends to explore the correlation between positive psychological quality and IPE and IEE for college students from the perspective of positive psychology in this study.

In summary, the feasibility of using positive psychology to analyze the integrated development of IPE and IEE of college students is high. Based on this, a questionnaire survey is investigated on 549 college students; the intermediary role of entrepreneurial self-efficacy in IEE, IPE in colleges, and entrepreneurial psychological quality of college students is analyzed using the linear regression analysis and hierarchical regression analysis; and integrated construction of IPE and IEE for college students under the positive psychology is evaluated comprehensively.

## Literature Overview

At present, applying positive psychology to discuss the entrepreneurial situation of college students has attracted more and more attentions of scholars. Chang et al. (2019) discussed the impacts and possible intermediary roles of network self-efficacy of non-information technology student on their core entrepreneurial capabilities, and found that the positive psychological thinking did regulate the correlation between the network entrepreneurial self-efficacy and network entrepreneurship intentions. Hu and Ye (2017) conducted a study on whether the entrepreneurial self-efficacies and entrepreneurial alertness of 364 Chinese students in sports majors could predict their entrepreneurial intentions, and found that the positive psychological reaction was a key cognitive predictor of self-efficacy, entrepreneurial alertness, and entrepreneurial intention of participant. Fernández-Pérez et al. (2019) analyzed the Spanish college students participating in compulsory entrepreneurship course by using the structural equation modeling and found that emotional ability had a positive impact on the formation of entrepreneurial intention and cognitive antecedents (entrepreneurial attitude and self-efficacy); students with higher emotional abilities who accept the entrepreneurship education had more positive attitudes toward entrepreneurship and believed that they were more capable to become entrepreneurs. Swartz et al. (2018) discussed the correlation between psychological independence and entrepreneurial intention and the importance of entrepreneurship of the college students, and found that psychological independence had a positive effect on entrepreneurship, but had no direct impact on the entrepreneurial intention; entrepreneurial spirit had a full intermediary role between the psychological independence and entrepreneurial intention. Auerbach et al. (2018) explored the social entrepreneurship intentions of high school and college students through the partial least squares structural equation model, and found that the correlation between self-efficacy and entrepreneurial intention was regulated by positive emotions and the perceived social support.

Mahendra et al. (2017) used the descriptive correlation design to analyze 230 students randomly selected from 540 students participating in three research projects, and found that the entrepreneurial intention was indirectly affected by entrepreneurial education, and the entrepreneurial motivation and attitude of the student were two important intermediary variables. Gozukara and Colakoglu (2016) explored the intermediary role of entrepreneurial alertness in the correlation between innovation ability and entrepreneurial intention of Turkish college students, and found that innovation had a positive impact on entrepreneurial intention and entrepreneurial alertness. Son et al. (2020) put forward the connotations and main characteristics of self-employment literacy of college students from the perspective of IPE, and expounded the practical value of self-employment literacy, aiming to help explore the development path of entrepreneurial quality of college students. Jun (2016) described the organic combination of IPE and IEE, indicating that the combination of the two was the key to meeting the comprehensive development needs of college students and improving their comprehensive abilities and qualities under the new situation.

In summary, there were many current researches on IEE of college students from the perspective of positive psychology, but there was less analysis on integration of IPE and IEE. Most of them were qualitative and lack of data discussion. Therefore, applying data quantitation for analysis of IPE and IEE is of great significance to cultivate the positive entrepreneurship psychological quality for college students.

## Methodology

### Research Objects

In this study, 549 college students from multiple colleges in the xxx area are selected as the research objects, with an average age of 19.41 ± 1.76 years and an age range of 17–23 years. A total of 600 questionnaires are distributed to all objects, and 549 are recovered. There are 537 valid questionnaires after excluding the ones with incomplete answers and invalid data, so the effective recovery rate is 89.5%.

**FIGURE 1:**
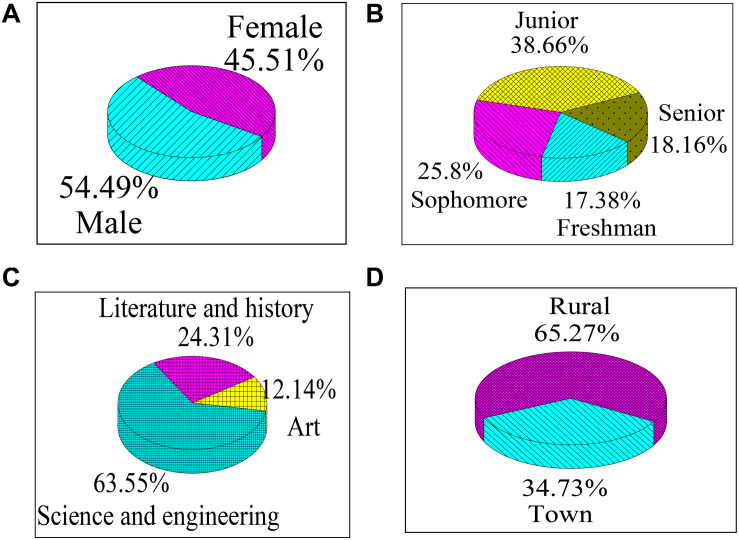
Demographic variables of the tested college students. **(A)** Indicates the gender ratio; **(B)** indicates the percentage of grade; **(C)** represents the percentage of major; and **(D)** represents the percentage of family location.

The demographic variables of the tested college students are given in Figure 1. The number of male college students (293) is slightly more than that of female college students (244), which is not greatly different. In terms of grades, the number of junior students is the largest (208), followed by sophomores (139) and seniors (98). Because juniors and seniors are about to graduate, they face greater employment pressure than freshmen and sophomores, so the sample selection has a certain bias. From the perspective of major, science students account for the largest proportion (341), followed by literature and history students (131). Besides, the colleges where the samples from are generally science and engineering colleges, so most samples are science and engineering students. In addition, the number of rural students (350) is significantly more than that of urban students (187), so rural students may have more entrepreneurial ideas compared with urban students with better family conditions.

### Hypotheses of the Study

In the current literature researches, they are basically independent analysis on IPE or IEE of college students, while the use of psychology in integrated research of the two-way construction of the two items is less. Therefore, the entrepreneurship psychological quality and entrepreneurial self-efficacy of college students are introduced in this study to analyze the correlation and adjustment mechanism between the IPE and IEE, providing further theoretical foundation for entrepreneurship education in colleges. Hypotheses proposed in this study are as follows:

H1: IPE of college students has a significantly positive impact on its positive entrepreneurship psychological quality.
H2: IEE of college students has a significantly positive impact on its positive entrepreneurship psychological quality.
H3: IPE of college students has a significantly positive impact on its entrepreneurial self-efficacy.
H4: IEE of college students has a significantly positive impact on its entrepreneurial self-efficacy.
H5: Entrepreneurial self-efficacy of college students has an intermediary role in correlation between IPE and positive entrepreneurship psychological quality.
H6: Entrepreneurial self-efficacy of college students has an intermediary role in correlation between IEE and positive entrepreneurship psychological quality.

### Questionnaire for Measuring

#### Questionnaires on Entrepreneurship Psychological Quality

A questionnaire on entrepreneurship psychological quality of college student is compiled by combing some difficulties of entrepreneurship and employment of college student, and referring to the predecessor entrepreneurship psychological quality scale (Yang and Ren, 2016). The questionnaire includes four aspects: entrepreneurial consciousness, entrepreneurial will, entrepreneurial ability, and entrepreneurial personality, with five items for each aspect. The Likert 5-point scale is adopted, with 1–5 indicating strong disagreement, disagreement, uncertainty, agreement, and strong agreement, respectively. The sum of the scores for the four aspects is the total score of the questionnaire. The higher the score, the better the enthusiasm of entrepreneurship psychological quality of college students, and it can be evaluated as the positive psychological quality if the score is higher than 60. The reliability and validity analysis shows that the internal consistency coefficients of the questionnaire and entrepreneurial awareness, entrepreneurial will, entrepreneurial ability, and entrepreneurial personality are 0.798, 0.852, 0.871, and 0.799, respectively.

#### IEE Questionnaires of College Students

Based on the establishment and development of IEE course in colleges, a questionnaire on IEE for college students is compiled in this study by referring to the innovation and entrepreneurship scale of college in predecessor literature (de Vibe et al., 2018). The questionnaire includes four dimensions: innovation and entrepreneurship course, innovation and entrepreneurship practice, innovation and entrepreneurship policy mastery, and innovation and entrepreneurship environment, with 5 items for each dimension. The Likert 5-point scale is used, with 1–5 indicating extreme non-conformance, non-conformance, uncertainty, conformance, and extreme conformance, respectively. The higher the score, the better the IEE course. According to the results of reliability and validity analysis, the internal consistency coefficients of the questionnaire and innovation and entrepreneurship course, innovation and entrepreneurship practice, innovation and entrepreneurship policy mastery, and innovation and entrepreneurship environment are 0.841, 0.802, 0.933, and 0.824, respectively.

#### Questionnaires on IPE of College Students

A questionnaire on IPE for college students is compiled based on the publicity work of major IPE in colleges and referring to the IPE scales in previous literatures (Dou et al., 2019). The questionnaire contains two main dimensions: main channels of IPE and daily IPE, with eight items for each dimension. The Likert 5-point scale is used, with 1–5 indicating extreme inconsistence, inconsistence, unknown result, consistence, and extreme consistence, respectively. The higher the score, the better the IPE effect of college students. The reliability and validity analysis shows that the internal consistency coefficients of the questionnaire and the main channels of IPE and daily IPE are 0.941 and 0.895, respectively.

#### Questionnaires on Entrepreneurial Self-Efficacy

Based on the self-efficacy theory and the definition of entrepreneurial self-efficacy of college students, a questionnaire on entrepreneurial self-efficacy of college students is compiled by referring to previous literatures (Cadenas et al., 2020). The questionnaire covers four dimensions: innovation efficiency, opportunity identification, relationship coordination, and organizational commitment, with four items for each dimension. The Likert 5-point scale is used, with 1–5 indicating strong disagreement, disagreement, uncertainty, agreement, and strong disagreement, respectively. The sum of the scores of the four dimensions is the total score of the questionnaire. The higher the score, the better the entrepreneurial self-efficacy of college students. The reliability and validity analysis shows that the internal consistency coefficients of the questionnaire and innovation efficiency, opportunity identification, relationship coordination, and organizational commitment are 0.911, 0.858, 0.926, and 0.803, respectively.

### Statistical Analysis

The data processing of this study is analyzed by SPSS19.0 version statistical software, and the measurement data is expressed by mean ± *SD* (x ± s). Spearman correlation and multiple regression are used to analyze the correlation between entrepreneurship psychological quality of college students, IEE in colleges, IPE, and entrepreneurial self-efficacy of college students. Hierarchical regression is used to analyze the intermediary role of entrepreneurial self-efficacy in IEE and IPE in colleges as well as entrepreneurship psychological quality. Origin 8.0 is used for graphing. *P* < 0.05 indicates the difference is statistically significant.

## Results

### Differences in Demographic Variables

**FIGURE 2:**
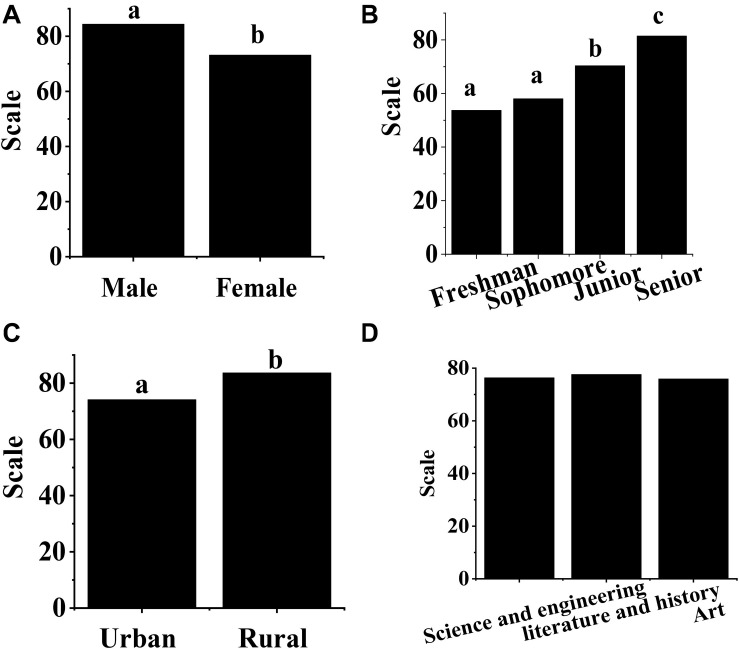
Differences in demographic variables of entrepreneurship psychological quality of college students. **(A–D)** show the comparisons on gender, grade, family location, and major, respectively. (a–c) indicate that the pair-comparison difference was statistically significant (*p* < 0.05).

As shown in Figure 2, there is a significant difference in the entrepreneurship psychological quality of college students in terms of gender, and the total score of entrepreneurship psychological quality of male college students (84.37 score) is significantly higher than that of female college students (73.15 score) (*p* < 0.05). The entrepreneurship psychological quality of college students shows great difference at the grade level, the total score of entrepreneurship psychological quality of senior students (81.45 score) is significantly higher than that of students in other grades (*p* < 0.05), and the total score of entrepreneurship psychological quality of senior students (70.36 score) is significantly higher than that of freshmen (53.71 score) and sophomores (58.03 score) (*p* < 0.05). No visible difference can be found in the total score of the entrepreneurial psychological quality of sophomore students compared with the freshman students (*p* > 0.05). There is a significant difference in entrepreneurship psychological quality of college students at the level of the family location, and the score of entrepreneurship psychological quality of rural students (83.63 scores) is obviously higher than that of urban college students (74.11 scores) (*p* < 0.05). There is no significant difference in entrepreneurship psychological quality of college students at the major level (*p* > 0.05).

### Correlation Between IPE and Positive Entrepreneurship Psychological Quality

As shown in Table 1, the main channels of IPE and daily IPE have significantly positive correlations with entrepreneurial awareness, entrepreneurial will, entrepreneurial personality, and total score of entrepreneurship psychological quality (*p* < 0.05), and have no significant correlation with the entrepreneurial ability (*p* > 0.05).

**TABLE 1:** Spearman correlation analysis on IPE and positive entrepreneurship psychological quality of college students.

Variable	Main channels of IPE		Daily IPE	
		
	*r*	*p*-value	*r*	*p*-value
Entrepreneurial awareness	5.828	0.015	5.117	0.008
Entrepreneurial will	4.722	0.009	4.280	0.018
Entrepreneurial ability	3.621	0.055	2.715	0.061
Entrepreneurial personality	6.378	0.042	5.185	0.021
Total score of entrepreneurship psychological quality	6.921	0.025	4.995	0.019

As shown in Table 2, to further analyze the predicative effect of IPE in positive entrepreneurship psychological quality of college students, the positive entrepreneurship psychological quality of college students is taken as dependent variable, and the main channels of IPE, daily IPE, and total score of IPE are undertaken as independent variables for linear regression analysis in this study. The results show that the regression coefficients between the main channels of IPE, daily IPE, and total score of IPE and the positive entrepreneurship psychological quality of college students is 0.519, 0.577, and 0.508, respectively, and they are significantly positively correlated (*p* < 0.05).

**TABLE 2:** Regression analysis on IPE and positive entrepreneurship psychological quality of college students.

Indicator	Regression coefficient	*T*-value	*p*-value
Main channels of IPE	0.519	4.628	0.025
Daily IPE	0.577	4.936	0.019
Total score of IPE	0.508	5.773	0.011

### Analysis on Correlation Between IEE and Positive Entrepreneurship Psychological Quality

Table 3 reveals that the entrepreneurial awareness, entrepreneurial will, entrepreneurial ability, entrepreneurial personality, and total score of entrepreneurial psychological quality have extremely significant correlations with innovation and entrepreneurship course, innovation and entrepreneurship practice, and innovation and entrepreneurship environment (*p* < 0.05), and have no significant correlation with innovation and entrepreneurship policy mastery (*p* > 0.05).

**TABLE 3:** Spearman correlation analysis on IEE and positive entrepreneurship psychological quality of college students.

Variable	Innovation and entrepreneurship course		Innovation and entrepreneurship practice		Innovation and entrepreneurship policy mastery		Innovation and entrepreneurship environment	
				
	*r*	*p*-value	*r*	*p*-value	*r*	*p*-value	*r*	*p*-value
Entrepreneurial awareness	5.911	0.019	6.954	0.004	2.657	0.053	4.517	0.011
Entrepreneurial will	4.946	0.021	5.678	0.017	3.196	0.062	5.362	0.031
Entrepreneurial ability	4.853	0.027	5.627	0.003	2.552	0.066	4.926	0.027
Entrepreneurial personality	6.008	0.038	5.316	0.017	3.166	0.082	3.967	0.038
Total score of entrepreneurship psychological quality	6.371	0.012	5.141	0.021	1.857	0.068	5.616	0.022

As shown in Table 4, to further analyze the predictive effect of IEE on the positive entrepreneurship psychological quality of college students, the positive entrepreneurship psychological quality of college students is taken as the dependent variable, and the innovation and entrepreneurship course, innovation and entrepreneurship practice, and innovation and entrepreneurship environment are undertaken as independent variables for linear regression analysis in this study. The results show that the regression coefficients between innovation and entrepreneurship course, innovation and entrepreneurship practice, innovation and entrepreneurship environment, and total scores of entrepreneurship and innovation education and the positive entrepreneurship psychological quality of college students are 0.710, 0.629, 0.557, and 0.701, respectively, and they have significantly positive effects (*p* < 0.05).

**TABLE 4:** Regression analysis on IEE and positive entrepreneurship psychological quality of college students.

Indicator	Regression coefficient	*T*-value	*p*-value
Innovation and entrepreneurship course	0.710	4.984	0.014
Innovation and entrepreneurship practice	0.629	5.626	0.031
Innovation and entrepreneurship environment	0.557	4.960	0.045
Total score of IEE	0.701	6.741	0.013

### Correlation Between IPE and Entrepreneurial Self-Efficacy

Table 5 indicates that the main channels of IPE and daily IPE under the IPE are significantly positively correlated with opportunity recognition, relationship coordination, organizational commitment, and self-efficacy (*p* < 0.05), and are not obviously correlated with innovation efficiency (*p* > 0.05).

**TABLE 5:** Spearman correlation analysis on IPE and entrepreneurial self-efficacy of college students.

Variable	Main channels of IPE		Daily IPE	
		
	*r*	*p*-value		*r*
Innovation efficiency	3.511	0.068	2.724	0.071
Opportunity recognition	5.378	0.036	4.116	0.039
Relationship coordination	6.106	0.016	5.742	0.008
Organizational commitment	5.721	0.014	5.007	0.023
Total score of self-efficacy	6.921	0.025	4.995	0.019

To further analyze the predictive effect of IPE on college students on the entrepreneurial self-efficacy, entrepreneurial self-efficacy of the college students is taken as the dependent variable, and the main channels of IPE, daily IPE, and the total score of IPE are taken as independent variables for linear regression analysis in this study. The results in Table 6 reveal that the regression coefficients between the main channels of IPE, daily IPE, and entrepreneurial self-efficacy are 0.622 and 0.536, respectively, and they are significantly positively correlated (*p* < 0.05); the regression coefficient between the total score of IPE and entrepreneurial self-efficacy is 0.722, which has an extremely positive correlation (*p* < 0.001).

**TABLE 6:** Regression analysis on IPE and entrepreneurial self-efficacy of college students.

Indicator	Regression coefficient	*T-*value	*p*-value
Main channels of IPE	0.622	5.026	0.008
Daily IPE	0.536	4.380	0.026
Total score of IPE	0.722	6.278	0.000

### Correlation Between IEE and Entrepreneurial Self-Efficacy

As shown in Table 7, the innovation and entrepreneurship course, innovation and entrepreneurship practice, and innovation and entrepreneurship environment have significantly positive correlations with innovation efficiency, opportunity recognition, organizational commitment, and total score of self-efficacy (*p* < 0.05), and have no significant correlation with relationship coordination (*p* > 0.05); the innovation and entrepreneurship policy mastery has no significant correlation with innovation efficiency, opportunity recognition, relationship coordination, organizational commitment, and total score of self-efficacy (*p* > 0.05).

**TABLE 7:** Spearman correlation analysis on IEE and entrepreneurial self-efficacy of college students.

Variables	Innovation and entrepreneurship course		Innovation and entrepreneurship practice		Innovation and entrepreneurship policy mastery		Innovation and entrepreneurship environment	
				
	*r*	*p*-value	*r*	*p*-value	*r*	*p*-value	*r*	*p*-value
Innovation efficiency	6.037	0.014	5.623	0.024	6.327	0.062	5.371	0.033
Opportunity recognition	4.011	0.026	5.211	0.039	5.426	0.057	4.719	0.016
Relationship coordination	2.621	0.061	2.448	0.058	4.520	0.081	2.882	0.057
Organizational commitment	5.701	0.031	4.952	0.021	2.115	0.055	6.001	0.024
Total score of self-efficacy	6.226	0.017	5.278	0.022	5.279	0.072	4.278	0.041

The entrepreneurial self-efficacy of college students is taken as the dependent variable, and the innovation and entrepreneurship course, innovation and entrepreneurship practice, innovation and entrepreneurship environment, and total score of IEE are taken as independent variables for linear regression analysis to further analyze the predictive effect of IEE on the entrepreneurial self-efficacy of college students. The results in Table 8 show that the regression coefficients between innovation and entrepreneurship course, innovation and entrepreneurship practice, innovation and entrepreneurship environment, and total scores of IEE and the entrepreneurial self-efficacy of college students are 0.630, 0.555, 0.498, 0.596, and 0.701, respectively, and they have significantly positive effects (*p* < 0.05).

**TABLE 8:** Regression analysis on IEE and entrepreneurial self-efficacy of college students.

Indicator	Regression coefficient	*T-*value	*p*-value
Innovation and entrepreneurship course	0.630	4.615	0.025
Innovation and entrepreneurship practice	0.555	5.482	0.018
Innovation and entrepreneurship environment	0.498	5.321	0.032
Total score of IEE	0.596	7.114	0.000

### Intermediary Role of Entrepreneurial Self-Efficacy in IPE and Positive Entrepreneurship Psychological Quality

Table 9 disclose that the entrepreneurial awareness, entrepreneurial will, entrepreneurial ability, and entrepreneurial personality have the significantly positive correlations with innovation efficiency, opportunity recognition, and organizational commitment (*p* < 0.05), and have no obvious correlation with relationship coordination (*p* > 0.05); the total score of entrepreneurship psychological quality has extremely obvious correlations with innovation efficiency, opportunity recognition, and organizational commitment (*p* < 0.001), and has no obvious correlation with relationship coordination (*p* > 0.05).

**TABLE 9:** Spearman correlation analysis on entrepreneurial self-efficacy and entrepreneurship psychological quality of college students.

Variables	Innovation efficiency		Opportunity recognition		Relationship coordination		Organizational commitment	
				
	*r*	*p*-value	*r*	*p*-value	*r*	*p*-value	*r*	*p*-value
Entrepreneurial awareness	4.831	0.021	7.112	0.008	2.412	0.052	5.615	0.021
Entrepreneurial will	5.715	0.033	5.717	0.015	3.005	0.054	6.052	0.026
Entrepreneurial ability	5.157	0.009	4.366	0.023	2.444	0.051	4.996	0.033
Entrepreneurial personality	5.737	0.026	5.641	0.016	3.215	0.050	3.752	0.030
Total score of entrepreneurship psychological quality	5.172	0.000	5.529	0.000	2.347	0.061	5.211	0.000

As shown in Table 10, the entrepreneurship psychological quality of college students is taken as dependent variable, and the innovation efficiency, opportunity recognition, organizational commitment, and total score of entrepreneurial self-efficacy are taken as independent variables for linear regression analysis, so as to further analyze the predictive effect of entrepreneurial self-efficacy on entrepreneurship psychological quality of college students. It results reveal that the regression coefficients between the innovation efficiency, opportunity recognition, organizational commitment, and the entrepreneurship psychological quality are 0.558, 0.472, and 0.664, respectively, and they have significant positive correlation (*p* < 0.05); the regression coefficient between the total score of entrepreneurial self-efficacy and entrepreneurship psychological quality is 0.577, which is extremely positively correlated (*p* < 0.001).

**TABLE 10:** Regression analysis on entrepreneurial self-efficacy and entrepreneurship psychological quality of college students.

Indicator	Regression coefficient	*T*-value	*p*-value
Innovation efficiency	0.558	4.852	0.021
Opportunity recognition	0.472	5.952	0.023
Organizational commitment	0.664	5.721	0.016
Total score of entrepreneurial self-efficacy	0.577	6.472	0.000

### Intermediary Role of Entrepreneurial Self-Efficacy in IEE and Positive Entrepreneurship Psychological Quality

Based on the aforementioned analysis results, the hierarchical regression analysis is used to analyze the intermediary role of entrepreneurial self-efficacy in IPE and entrepreneurship psychological quality. As shown in Tables 11, 12, the standardized regression equations tested in sequence are significant at the level of *p* < 0.05, and the intermediary role of entrepreneurial self-efficacy accounts for 33.49%.

**TABLE 11:** Regression analysis on IPE and entrepreneurship psychological quality.

Indicator		Standardized regression coefficient	*T*-value	*p*-value	*R*^2^	*F*-value
Dependent variable	IPE	0.538	6.117	0.012	0.191	250.61
Intermediary variable	Entrepreneurial self-efficacy	0.497	5.362	0.026		

**TABLE 12:** The hierarchical test of entrepreneurial self-efficacy in IPE and entrepreneurship psychological quality.

Indicator		Step 1	Step 2	Step 3
Standardized regression equation		Y = 0.510X	M = 0.412X	Y = 0.296M + 0.335X
Test of regression coefficient	SE	0.041	0.027	0.031
	*t*	5.769*	6.577*	4.783*

*Suggested the difference was statistically significant (p < 0.05).

The intermediary role of entrepreneurial self-efficacy in IEE and entrepreneurship psychological quality are analyzed with the hierarchical regression analysis in this study. Tables 13, 14 reveal that the standardized regression equations are significant at the level of *P* < 0.05, and the intermediary role of entrepreneurial self-efficacy accounts for 41.85%.

**TABLE 13:** Regression analysis on IEE and entrepreneurship psychological quality.

Indicator		Standardized regression coefficient	*T*-value	*p*-value	*R*^2^	*F-*value
Dependent variable	IEE	0.618	5.716	0.009	0.316	189.52
Intermediary variable	Entrepreneurial self-efficacy	0.617	5.367	0.017		

**TABLE 14:** The hierarchical test of entrepreneurial self-efficacy in IEE and entrepreneurship psychological quality.

Indicator		Step 1	Step 2	Step 3
Standardized regression equation		Y = 0.510X	M = 0.412X	Y = 0.296M + 0.335X
Test of regression coefficient	SE	0.041	0.027	0.031
	*t*	5.769*	6.577*	4.783*

*Suggested the difference was statistically significant (p < 0.05).

**FIGURE 3:**
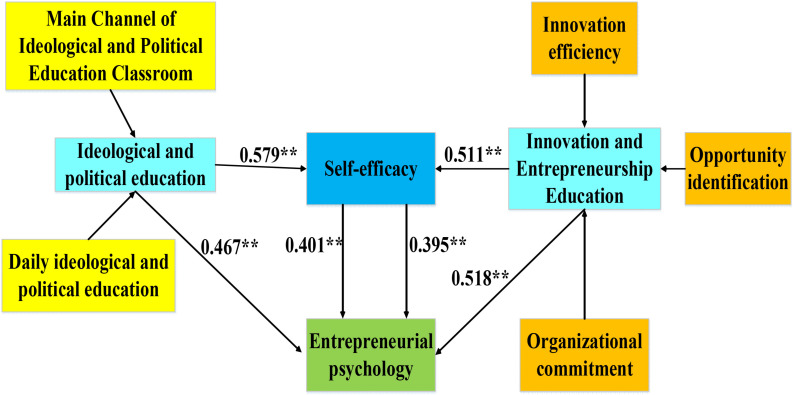
Model on correlation between entrepreneurial self-efficacy in integrated construction of IPE and IEE and the entrepreneurship psychological quality. ^∗∗^*p* < 0.001.

Based on the aforementioned analysis of intermediary roles, the model on correlation between entrepreneurial self-efficacy in integrated construction of IPE and IEE and the entrepreneurship psychological quality can be constructed (Figure 3).

## Discussion

The development of IEE in China has been in full swing in recent years, and colleges around the country have actively responded to the national issue of the exhibition IEE course. It has promoted the construction of entrepreneurial activities for college students to a certain extent, but it is still on the surface of education, the effective integration of IEE and IPE is not achieved, and the advantage of ideological and political workers being familiar with the characteristics of students is not used to enhance the educational effect (Noh et al., 2019). Therefore, 549 college students from multiple colleges in the xxx area are selected for in-depth discussion in this study. It is found that there is a significant difference in the entrepreneurship psychological quality of college students at the level of gender, and the total score of entrepreneurship psychological quality of male college students is significantly higher than that of female college students (*p* < 0.05), which is different from the research results of Chen (2019). Such difference may be because it is generally believed that males should be more innovative and entrepreneurial, and higher requirements are given for male innovation and entrepreneurship training, and they will also receive more support from families (Nikiforou et al., 2019). There is a significant difference in the entrepreneurship psychological quality of college students at the grade level, and the total score of entrepreneurship psychological quality of senior students is significantly higher than that of students in other grade (*p* < 0.05), As seniors face greater employment and entrepreneurial pressure, they are cultivated with more IEE, which can improve their positive psychological quality. There is a significant difference in entrepreneurship psychological quality of college students at the level of the family location, and the score of entrepreneurship psychological quality of rural students is significantly higher than that of urban college students (*p* < 0.05), which is similar to the research results of Yu et al. (2016). Rural students may have more entrepreneurial ideas compared with urban students with better family conditions.

With Spearman correlation and multiple regression analysis, it can be known that the regression coefficients of the main channels of IPE, daily IPE, total scores of IPE, and positive psychological quality of undergraduate entrepreneurship are 0.519, 0.577, and 0.508, respectively, which all have significant positive impacts on positive psychological quality (*p* < 0.05). It indicates that the implementation of IPE in colleges can significantly improve the entrepreneurial awareness and entrepreneurial will of college students, so H11 is true. The main channels of IPE and daily IPE have significantly positive effects on the entrepreneurial self-efficacy (*p* < 0.05); the total score of IPE has an extremely significantly positive effect on the entrepreneurial self-efficacy (*p* < 0.001), which is similar to the results of McCarthy et al. (2018), suggesting that IPE has a significant impact on the cultivation of innovation efficiency and opportunity recognition ability of college students, so the H3 is true (Hu, 2016). Innovation and entrepreneurship course, innovation and entrepreneurship practice, innovation and entrepreneurship environment, and total score of IEE have significantly positive effects on positive entrepreneurship psychological quality and entrepreneurial self-efficacy of college students (*p* < 0.05), suggesting that the IEE can effectively promote the cultivation of positive entrepreneurship psychological quality and entrepreneurial self-efficacy, so the H2 and H4 are true (Pearson et al., 2017). It can be known by using the hierarchical regression analysis that the intermediary roles of entrepreneurial self-efficacy in IPE and entrepreneurship psychological quality are 33.49%, and that in IEE and entrepreneurship psychological quality is 41.85%, which is similar to the findings of Basinska and Dåderman (2018). Entrepreneurial self-efficacy plays a full intermediary role in IPE, IEE, and entrepreneurship psychological quality, so the H5 and H6 are true. The integrated construction of IPE and IEE can improve the innovation efficiency of college students and establish the positive entrepreneurship psychological quality.

## Conclusion

In this study, the integrated construction and application of IPE and IEE in colleges based on the positive psychological quality of entrepreneurship are analyzed with the hierarchical regression test. The results reveal that the entrepreneurship psychological quality of college students differs significantly in gender, family location, and grade. Entrepreneurial self-efficacy plays a full intermediary role in IPE, IEE, and entrepreneurship psychological quality. The integrated construction of IPE and IEE can improve the innovation efficiency of college students, and cultivate the positive entrepreneurship psychological quality. However, due to the limitation of practical conditions, the size of college students in this study is small, which has certain regional limitations and common general promotion. It will consider increasing the selection of student samples and introducing the entrepreneurship teaching of university teachers to further explore the correlation between the IPE and entrepreneurship education for college students. In conclusion, it provides a theoretical basis for the joint development of IPE and IEE for college students.

## Data Availability Statement

The raw data supporting the conclusions of this article will be made available by the authors, without undue reservation.

## Ethics Statement

The studies involving human participants were reviewed and approved by the Northwestern Polytechnical University Ethics Committee. The patients/participants provided their written informed consent to participate in this study. Written informed consent was obtained from the individual(s) for the publication of any potentially identifiable images or data included in this article.

## Author Contributions

Both authors listed have made a substantial, direct and intellectual contribution to the work, and approved it for publication.

## Conflict of Interest

The authors declare that the research was conducted in the absence of any commercial or financial relationships that could be construed as a potential conflict of interest.
